# Metachronous skeletal muscle metastasis without any other organ metastases after curative gastrectomy: a case report

**DOI:** 10.1186/s40792-018-0507-3

**Published:** 2018-08-17

**Authors:** Naoki Kamitani, Akihiko Watanabe, Yuki Kirihataya, Saiho Ko

**Affiliations:** 1 Department of Surgery, Nara Prefectural General Medical Center, 1-30-1 Hiramatsu, Narashi, Nara 631-0846 Japan; 2 Department of Surgery, Nara Medical University, 840 Shijocho, Kashiharashi, Nara 634-8522 Japan

**Keywords:** Gastric cancer, Skeletal muscle metastasis, Chemotherapy

## Abstract

**Background:**

Skeletal muscle metastasis from gastric cancer is extremely rare and often accompanied with synchronous metastasis to any other organs. We herein report a case of rapidly developing multiple skeletal metastases from gastric cancer without any other organ metastases.

**Case presentation:**

A 47-year-old man underwent distal gastrectomy for advanced gastric cancer. Pathological diagnosis was poorly differentiated adenocarcinoma, T2N1M0, Stage IIA. The patient presented with a history of left dorsal tenderness 12 months after the operation. A computed tomography (CT) revealed a solid mass in the left latissimus dorsi muscle. Pathological examination of the ultrasound guided needle biopsy specimen revealed poorly differentiated adenocarcinoma similar to the previously resected gastric cancer, and the tumor was diagnosed as metastasis of gastric cancer. Thereafter, the systemic chemotherapy was administrated. However, the metastases were extended to the paraspinal muscle and quadriceps, and the patient died 7 months after the recurrence.

**Conclusions:**

The prognosis of patients with skeletal muscle metastasis may be extremely poor, even in patients without any other organ metastases. The development of further chemotherapeutic agents and regimens is therefore needed.

## Background

Surgery is the mainstay of treatment for gastric cancer. However, an appreciable proportion of patients with advanced gastric cancer develop recurrence, even after curative resection. The common sites of recurrence and metastasis were lymph nodes, peritoneum, and liver [1]. On the other hand, skeletal muscle metastasis from gastric cancer is extremely rare, and prognosis of patients with such metastasis has been reported to be very poor [1]. We herein report a case of rapidly developing multiple skeletal metastases from gastric cancer without any other organ metastases.

## Case presentation

**Fig. 1:**
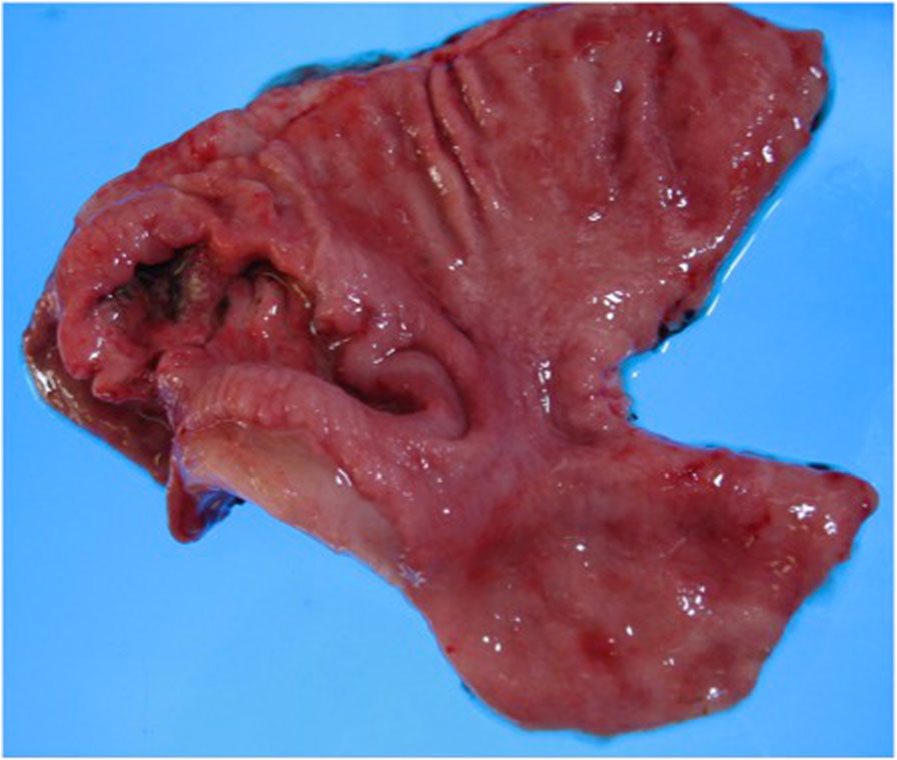
Resected specimen. Resected specimen showed a circumferential type 3 tumor at the pyloric antrum of the stomach

**Fig. 2:**
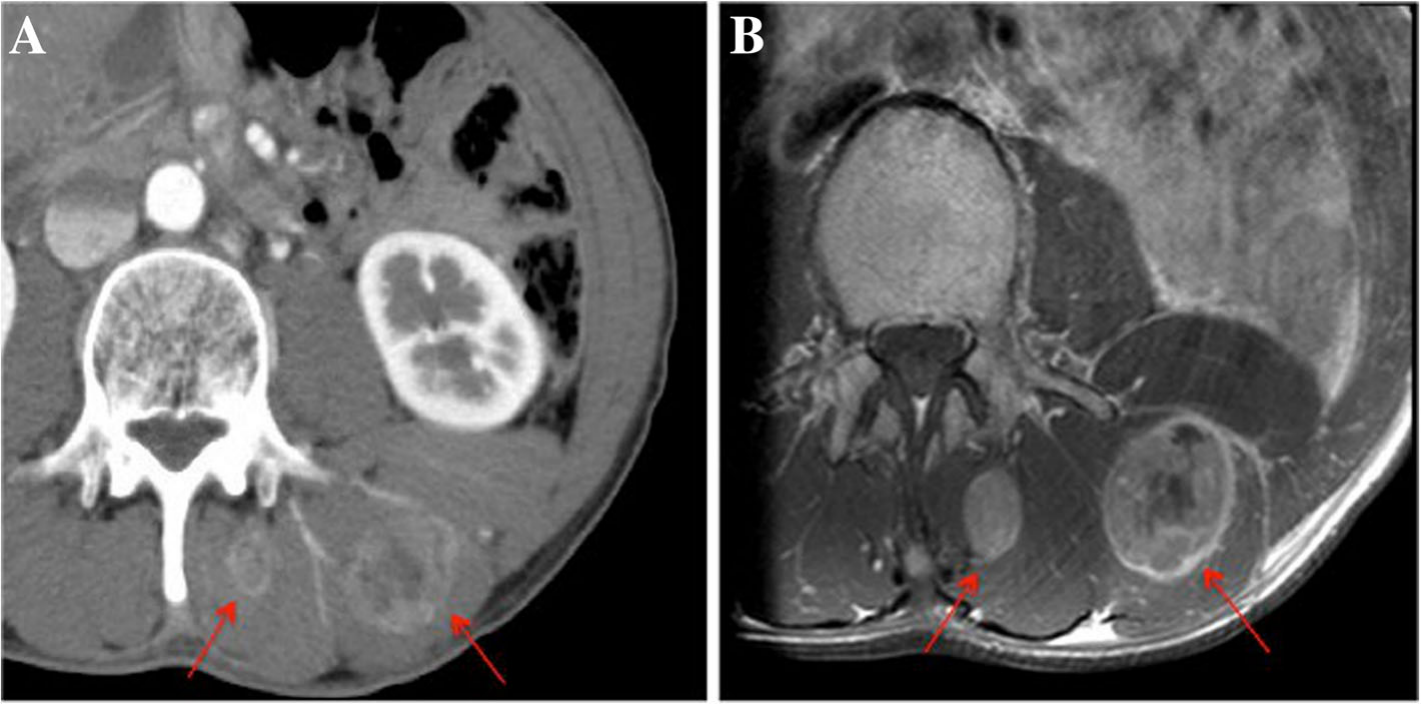
**a** An abdominal computed tomography scan revealed a solid mass (arrows), measuring 40 mm × 30 mm, in the left latissimus dorsi muscle. **b** A contrast-enhanced magnetic resonance imaging showed extensive peritumoral enhancement (arrows)

**Fig. 3:**
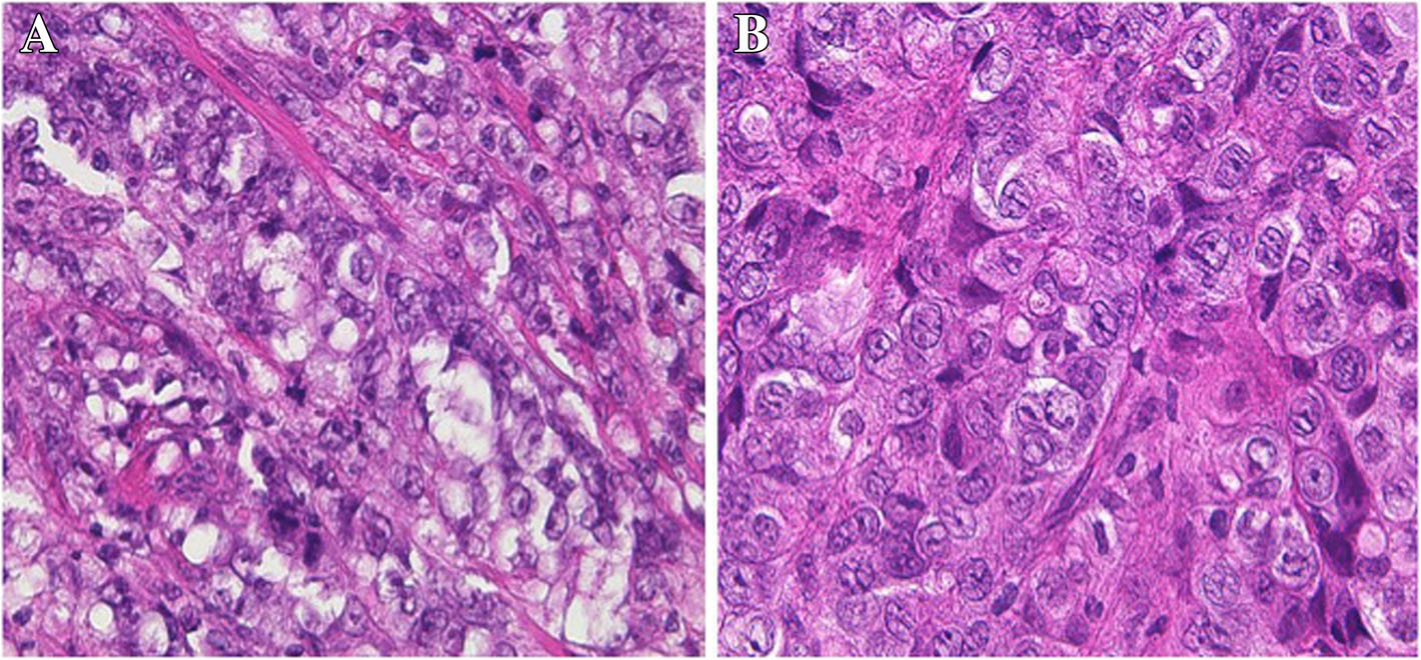
**a** Pathological examination of primary gastric cancer revealed poorly differentiated adenocarcinoma (H.E. stain × 400). **b** Pathological examination of the tumor in left latissimus dorsi muscle revealed poorly differentiated adenocarcinoma, similar to the previously resected gastric cancer (H.E. stain × 400)

**Fig. 4:**
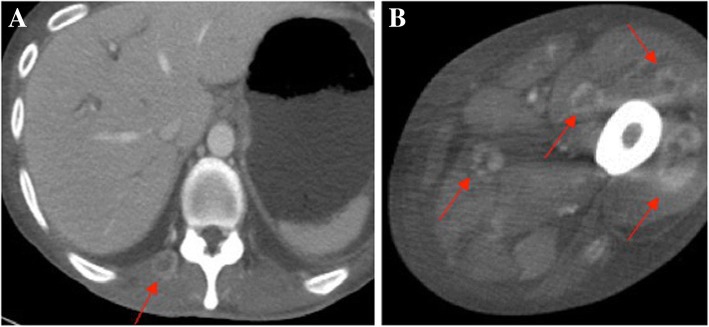
The metastases was extended to paraspinal (**a**) and quadriceps muscle (**b**) (arrows)

A 47-year-old man underwent distal gastrectomy with D2 lymph node resection for gastric cancer. Resected specimen disclosed a circumferential type 3 tumor at the pyloric antrum of the stomach (Fig. 1). Pathological diagnosis based on the third English edition of the Japanese classification of gastric carcinoma was poorly differentiated adenocarcinoma, pT2, ly2, v1, pN1, pM0, and pStage IIA. Then, the patient underwent adjuvant chemotherapy of S-1. CT at 11 months after the operation revealed no recurrence. The patient presented with left dorsal tenderness 12 months after the operation, and CT revealed a solid mass, measuring 40 mm × 30 mm, in the left latissimus dorsi muscle (Fig. 2a). Contrast-enhanced magnetic resonance imaging (MRI) revealed extensive peritumoral enhancement (Fig. 2b). A positron emission tomography (PET) revealed elevated [18F]-fluorodeoxyglucose uptake in the tumor. CT, MRI, and PET did not reveal any other metastases. We then performed the ultrasound guided needle biopsy of the tumor. Pathological examination of the biopsy specimen revealed poorly differentiated adenocarcinoma similar to the previously resected gastric cancer (Fig. 3a and b), and the tumor was diagnosed as metastasis of gastric cancer. Thereafter, the patient underwent two courses of a combination chemotherapy of S-1 and cisplatin, two courses of S-1 and docetaxel, and one course of S-1 and CPT-11. However, all of chemotherapeutic regimens were not effective, and the metastases were extended to the paraspinal muscle and quadriceps 6 months after the recurrence (Fig. 4a and b). The patient died 7 months after the recurrence. Autopsy was not performed. The pain from muscle metastasis was under control with oral non-steroidal anti-inflammatory drugs, oral oxycodone and/or transdermal fentanyl.

The liver, peritoneum, and lymph nodes are common metastatic sites for gastric cancer. The muscle is well vascularized, and hematogenous metastasis is therefore expected to occur in muscular tissue; however, it has been reported that skeletal muscle metastasis rarely occurs in gastric cancer patients, with the incidence rate of 0.03–0.16% [2–4]. Although the reason for the rarity of skeletal muscle metastasis is unclear, the following reasons have been suggested: (1) the prevention of settlement of tumor cells by the inconstant blood flow and changing tissue pressure due to muscular contractions, (2) the inhibition of tumor cell proliferation by lactic acid, and (3) lower PH values in the muscle [5]. Skeletal muscle metastasis is generally associated with widespread metastatic disease, and often accompanied with synchronous metastasis to any other organs [5]. Skeletal muscle metastasis without any other organ metastases is therefore extremely rare. The present case had normal level of exercise, and the reason why muscle metastasis without any other organ metastases occurred in the present case remains uncertain.

It is sometimes difficult to distinguish muscle metastasis from primary soft tissue sarcoma accurately. Needle or open biopsy is usually performed to confirm a diagnosis. The imaging modalities, such as CT, MRI, ultrasonography, and gallium scintigraphy, have been employed to make a diagnosis. MRI has been reported to be the most useful not only for differential diagnosis but also for delineating the extent of the involved muscle, because of its high contrast to the surrounding muscle and its wider observational capability via multiple sections [1]. Muscle metastasis typically shows low- to iso-signal intensity on T1 weighted images and high-signal intensity on T2 weighted images compared with surrounding muscle. However, primary soft tissue sarcoma can show the similar findings. Contrast-enhanced MRI reveals extensive peritumoral enhancement and lobulation associated with central necrosis in muscle metastasis. These findings seem to be useful to distinguish between muscle metastasis and primary soft tissue sarcoma [1]. However, the histological examination should be performed to make the definitive diagnosis of muscle metastasis. In our case, the ring-enhanced peritumoral area on MRI suggested the skeletal muscle metastasis, and the definitive diagnosis was able to be achieved by the needle biopsy. Immunohistochemical examination is useful for the definitive diagnosis, although data on immunohistochemical staining is unavailable in our case. In general, gastric cancer cell shows positive staining for cytokeratin 7 [5], and desmin and muscle specific actin are positive in primary soft tissue sarcoma [6].

We reviewed the reported cases of skeletal muscle metastasis from gastric cancer. To search the literature, we used key words of “gastric cancer (or carcinoma)” and “muscle (or musclar) metastasis (or metastases)”, and found five cases that had metachronous skeletal muscle metastasis without any other organ metastases after curative gastrectomy (Table 1) [2, 7–10]. In detail, all cases were male, and median age was 60.5 years (range 47–75 years). Median duration between gastrectomy and muscle metastasis was 14 months (range 7–60 months). Four cases had multiple muscle metastases, and the remaining two cases had solitary metastasis. The histologic type was described in four cases, and all of these four cases had undifferentiated adenocarcinoma. The stage of gastric cancer was described in four cases, including three cases of stage IIA and one case of stage IB. These findings suggest that skeletal muscle metastasis without any other organ metastases tend to occur in patients with relatively early stage disease and undifferentiated adenocarcinoma.

**Table 1:** Review of the six reported cases of metachronous skeletal muscle metastasis after curative gastrectomy

Case	Year	Author	Age (years)	Sex	Time after the operation (months)	Histologic type	Stage	No. of metastasis
1	1993	Sudo	61	M	60	Adenocarcinoma	N/A	Solitary
2	1996	Amano	57	M	7	Adenocarcinoma	N/A	Multiple
3	1997	Ghanekar	75	M	15	por	IIA (T2N1M0)	Solitary
4	2006	Beşe	60	M	13	sig	IIA (T3N0M0)	Multiple
5	2015	Koga	71	M	36	por	IB (T2N0M0)	Multiple
6	2018	Ours	47	M	12	por	IIA (T2N1M0)	Multiple

*M* male*, por* poorly differentiated adenocarcinoma, *sig* signet ring cell carcinoma, *N/A* not available

Among the six reported cases, surgical excision was performed in only one case with solitary muscle metastasis (Table 2). Surgical excision was reported to relieve the pain due to the muscle metastasis [5]. Radiotherapy may also be effective in relieving pain and reduction in the size of metastatic lesions [5]. Chemotherapy is considered as the standard treatment for the muscle metastasis of gastric cancer. Four cases, including the present case, underwent chemotherapy. These included doxorubicin, a combination chemotherapy of 5-fluorouracil, leucovorin, and cisplatin, a combination chemotherapy of S-1 and cisplatin, chemotherapy plus trastuzumab. A phase III trial demonstrated that the overall survival was better in patients with advanced gastric cancer treated with S-1 and cisplatin than with S-1 alone [11]. Based on the results of this trial, the Japanese gastric cancer treatment guidelines recommend a combination of S-1 and cisplatin as the first-line chemotherapy for recurrent gastric cancer [12]. In the present case, the pain had been manageable with an oral analgesic. Therefore, we firstly performed a combination chemotherapy of S-1 and cisplatin, and the present case was the only case that underwent S-1-based chemotherapy among the reported cases.

**Table 2:** Treatment and outcomes of the six reported cases of metachronous skeletal muscle metastasis after curative gastrectomy

Case	Treatment	Chemotherapeutic regimen	Prognosis (months)
1	CRT	Doxorubicin	Dead (6)
2	N/A	–	Dead (N/A)
3	Surgery	–	N/A
4	CRT	5-FU + leucovorin + cisplatin	Alive (24)
5	Chemotherapy	Chemotherapy + trastuzumab	Dead (18)
6	Chemotherapy	S-1 + cisplatin	Dead (7)

*CRT* chemoradiotherapy, *N/A* not available, *5-FU* 5-fluorouracil

The prognosis of patients with skeletal muscle metastasis was reported to be extremely poor. Among the reported cases, four cases had died, with a median survival time of 12.5 months (range 6–18 months). Only one case, which underwent chemoradiotherapy for multiple skeletal metastases, remained alive at 24 months after recurrence [2]. The metastasis to skeletal muscle seems to be a sign of systemic hematogenous metastasis and the terminal stage in the progress of gastric carcinoma [1]. Although the present case underwent a S-1-based chemotherapy, including S-1 and cisplatin, S-1 and docetaxcel, and S-1 and CPT-11, all of these regimens were not effective, and the patient died 7 months after the recurrence. More recently, some studies suggest that S-1-containing chemotherapy is ineffective in patients who showed S-1 adjuvant failure [13]. In addition, no benefit of adding S-1 beyond progression has been shown in a recent study. On the other hand, several molecularly targeted drugs such as trastuzumab, ramucirumab, and nivolumab have been developed, and the efficacy of such drugs has been shown in clinical trials [14–16]. Therefore, a change of the key drug and molecularly targeted drugs are expected to prolong survival time of patients with skeletal muscle metastasis of gastric cancer.

## Conclusions

Herein, we reported a very rare case with skeletal muscle metastasis of gastric cancer without any other organ metastases. The prognosis of patients with skeletal muscle metastasis may be extremely poor, even in patients without any other organ metastases.

## Abbreviations

CT: Computed tomography
MRI: Magnetic resonance imaging
PET: Positron emission tomography
